# Achieving enhanced cell penetration of short conformationally constrained peptides through amphiphilicity tuning†
†Electronic supplementary information (ESI) available. See DOI: 10.1039/c7sc03614k


**DOI:** 10.1039/c7sc03614k

**Published:** 2017-09-13

**Authors:** Yuan Tian, Xiangze Zeng, Jingxu Li, Yanhong Jiang, Hui Zhao, Dongyuan Wang, Xuhui Huang, Zigang Li

**Affiliations:** a School of Chemical Biology and Biotechnology , Shenzhen Graduate School of Peking University , Shenzhen , 518055 , China . Email: lizg@pkusz.edu.cn; b School of Life Science and Engineering , Southwest Jiaotong University , Chengdu , 610031 , China; c Department of Chemistry , Center of Systems Biology and Human Health , School of Science and Institute for Advance Study , The Hong Kong University of Science and Technology , Clear Water Bay , Kowloon , Hong Kong . Email: xuhuihuang@ust.hk

## Abstract

Due to their enhanced stability and cell permeability, cyclic cell-penetrating peptides have been widely used as delivery vectors for transporting cell-impermeable cargos into cells. In this study, we synthesized a panel of conformationally constrained peptides with either α-helix or β-hairpin conformations. We tuned the amphiphilicity of these constrained peptides with different distributions of charged or hydrophobic residues and compared their cellular uptake efficiencies in different cell lines. We found that the amphipathicity of these conformationally constrained peptides correlates well with their cellular uptake efficiency. We proposed that peptides with larger hydrophobic moments (HMs) have stronger binding affinities with the cell membrane which further accelerates the endocytosis process. This finding should provide an approach towards the design of more potent conformationally constrained cell-penetrating peptides for biomedical applications.

## Abstract 2

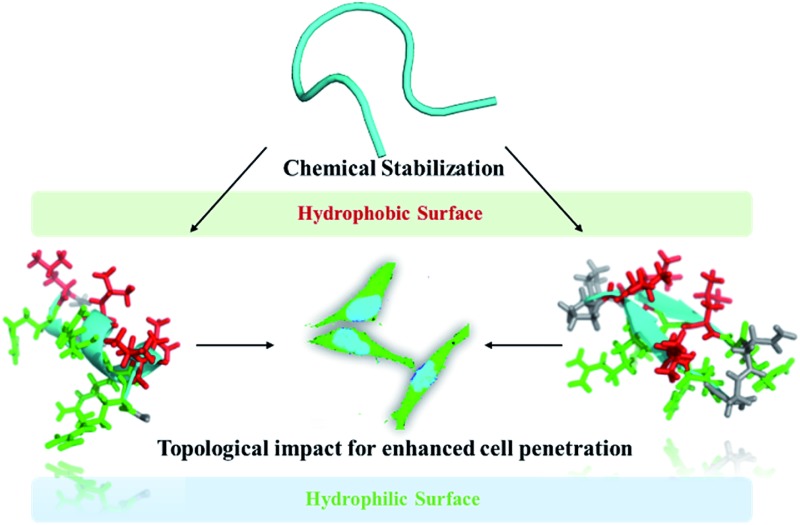
We synthesized a panel of conformationally constrained peptides with either α-helix or β-hairpin conformations. We tuned the amphiphilicity of these constrained peptides with different distributions of charged or hydrophobic residues and compared their cellular uptake efficiencies in different cell lines.

## Introduction

**Fig. 1:**
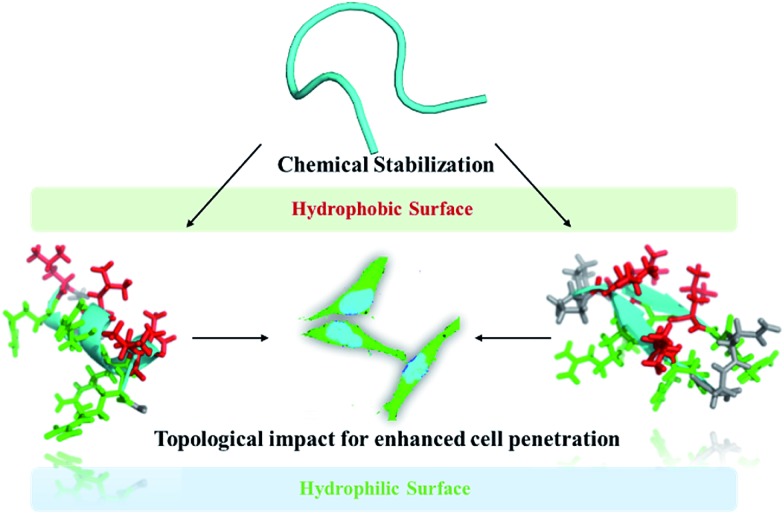
Schematic illustration of the topological impact of short conformationally constrained cell-penetrating peptides for enhanced cell penetration.

Cell-penetrating peptides (CPPs) are a class of short cationic peptide that are capable of crossing biological membranes and transporting other cell-impermeable molecular cargoes into cells.^1^–^5^ Since the first observation of the HIV-1 transactivator protein (Tat) crossing the cell membrane in 1988, CPPs have been initially defined as protein transduction domain (PTD) sequences, abundant with positively charged amino acids such as lysine and arginine.^6^–^9^ The guanidinium-rich Tat peptides were broadly utilized for delivering a variety of cargoes into cells. The conjugation of molecular cargoes to other linear CPPs has also been extensively studied.^10^–^12^ Compared to linear peptides, cyclic peptides have drawn significant interest as they feature improved proteolytic stability, and the rigidified peptide backbone offers enhanced cell permeability.^13^–^15^ In 2011, Parang *et al.* reported a series of homochiral cyclic peptides as alternative highly stable nuclear-targeting molecular transporters.^16^–^18^ In 2014, cyclic Tat peptides were reported to be capable of delivering GFP proteins with immediate bioavailability.^19^ Another previous study demonstrated that the maximal separation of guanidinium groups in arginine-rich peptides through cyclization facilitates their cellular uptake efficiency.^20^ In particular, Pei *et al.* reported a family of amphipathic cyclic peptides with up to 120% cytosolic delivery efficiencies compared to those of the Tat sequence.^21^–^23^ These cyclic CPPs bind directly to the plasma membrane phospholipids before entering mammalian cells. Their cellular uptake efficiency correlates positively with the binding affinity for the membrane components. Furthermore, they elucidated a novel mode for the endosome escape of their cyclic CPPs, in which the peptides could induce membrane curvature and the budding of small vesicles, which eventually collapsed and aggregated. Controlling the charge display from helical backbones was previously reported as another feasible strategy to enhance the cellular uptake of cell-penetrating peptides. A study by Schepartz and colleagues demonstrated that a specific arginine topology is crucial for helical mini-proteins to escape from the endosomes and be released into cytosol.^24^,^25^ Gellman *et al.* investigated the effects of conformational stability and the geometry of the guanidinium display on the cell-penetrating properties of helical β-amino acid oligomers.^26^,^27^ Recently, Wennemers *et al.* investigated the effect of preorganized charge display on the cellular uptake of the guanidinylated polyproline II (PPII) helix.^28^ In order to further diversify the patterns of charge display in rigid scaffolds, in this study, we set out to reinforce short peptides into either rigidified α-helix or β-hairpin conformations with different geometries of guanidinium display, and studied their cell-penetrating properties. This conformational difference led to distinct cellular uptakes and should further guide the search for even more potent constrained cell-penetrating peptides (Fig. 1).

## Results and discussion

**Fig. 2:**
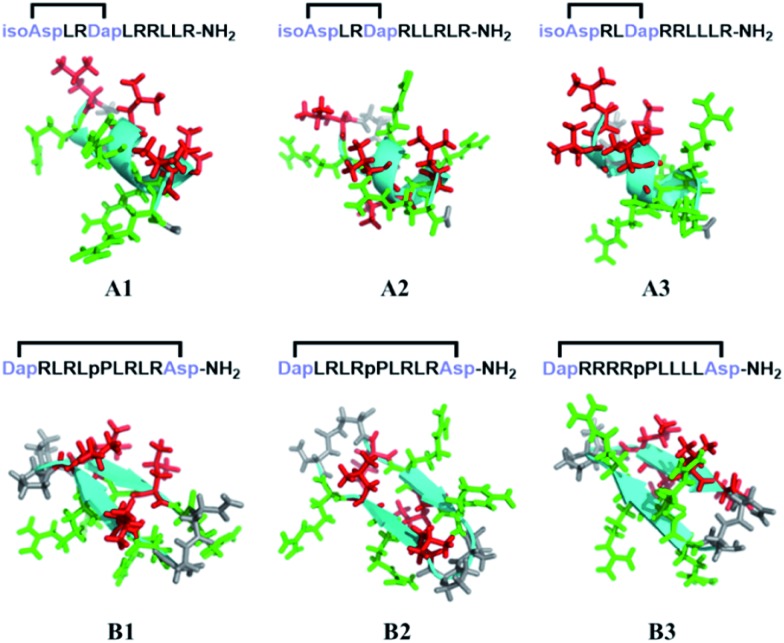
Molecular three-dimensional structure projections of conformationally constrained amphipathic cell-penetrating peptides with different topological distributions of hydrophilic and hydrophobic residues. Hydrophobic residues are shown in red, hydrophilic residues are shown in green and backbones are shown in cyan.

Linear CPPs are generally unstructured in solution, and thus it remains an open question as to how specific conformations influence the cellular uptake of constrained cell-penetrating peptides. In addition, different conformations in turn lead to entirely different topological distributions of positively charged residues and hydrophobic residues, which we believe should play an important role in interacting with and crossing biological barriers. A number of chemical approaches have been reported to successfully constrain unstructured peptides into protein secondary structural elements, including peptide stapling, template nucleation and diaminodiacid-based macrocyclization.^29^–^32^ Our previous work has demonstrated an efficient helix nucleation strategy using terminal diacid as a helix inducer.^33^,^34^ This method could efficiently constrain short peptides into α-helical conformations. On the other hand, using turn mimetics by introducing β-hairpin inducers followed by macrocyclization is the most widely used strategy to constrain peptides into β-hairpin conformations.^35^–^37^ d-Pro-l-Pro is one of the most established templates. Herein, we set out to utilize the above two nucleation templates with the same macrolactamization chemistry to access amphipathic cell-penetrating peptides in both α-helix and β-hairpin conformations with different topological distributions of hydrophilic and hydrophobic residues, and study their cellular uptakes in different cell lines. The design of the constrained peptides was based on the different topological distributions of hydrophilic arginine and hydrophobic leucine. Molecular three-dimensional structure projections of the peptides are shown in Fig. 2. Peptide A1 was designed to possess a classical amphipathic pattern with arginine on one face of the helix barrel and leucine on the other face. Peptide A2 possesses a mixed distribution of hydrophobic and hydrophilic residues. The hydrophilic and hydrophobic residues of peptide A3 were designed to be distributed at the N-terminus and C-terminus of the peptide. For peptides with β-hairpin conformations, B1 displayed a similar distribution to peptide A1, with arginine and leucine at two faces of the sheet. B2 possesses a mixed distribution and B3 is designed with arginine positioned at one side of the sheet backbone. The peptides were synthesized through standard solid phase peptide synthesis and cyclized through macrocyclization. All of the syntheses were performed on resin, as shown in the ESI.†

**Fig. 3:**
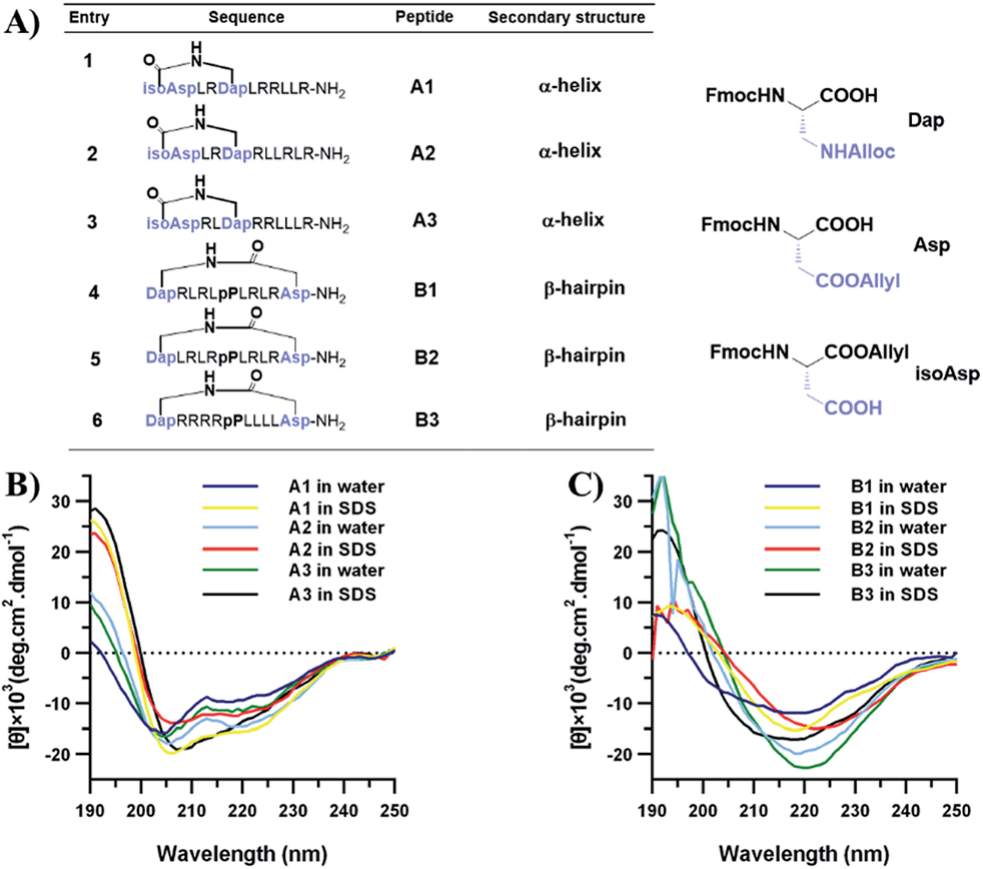
Peptide sequences and conformational analysis of the constrained peptides. (A) The peptide sequences and unnatural amino acids used in this study. (B) The CD spectra of α-helical peptides in water and 25 mM SDS. (C) The CD spectra of β-hairpin peptides in water and 25 mM SDS.

To confirm the conformational preference of our designed constrained peptides experimentally, we characterized the secondary structures of each peptide both in deionized water and 25 mM sodium dodecyl sulfate (SDS) using circular dichroism (CD) spectroscopy (Fig. 3). An anionic surfactant such as SDS was previously used to mimic the cell membrane environment.^38^,^39^ The CD spectra show that our synthesized short constrained peptides form well-defined secondary structures. For the helix-promoting A-series peptides stabilized by a terminal diacid template, the CD spectra exhibit positive peaks at around 190 nm and two negative minimum bands at 208 and 222 nm, indicating the predominance of α-helix structures. On the other hand, the CD spectra of the B-series peptides show a substantial difference, marked by the intensification of the negative peak at around 215–220 nm, which is characteristic of the β-hairpin conformation. In the membrane-mimicking environment, both the helix-promoting and hairpin-promoting peptides adopted similar conformations to those in deionized water, which indicates that the secondary structures of these short cyclic peptides were effectively fixed in the desired conformations using the two template strategies, and that the conformations remained relatively rigid.

**Fig. 4:**
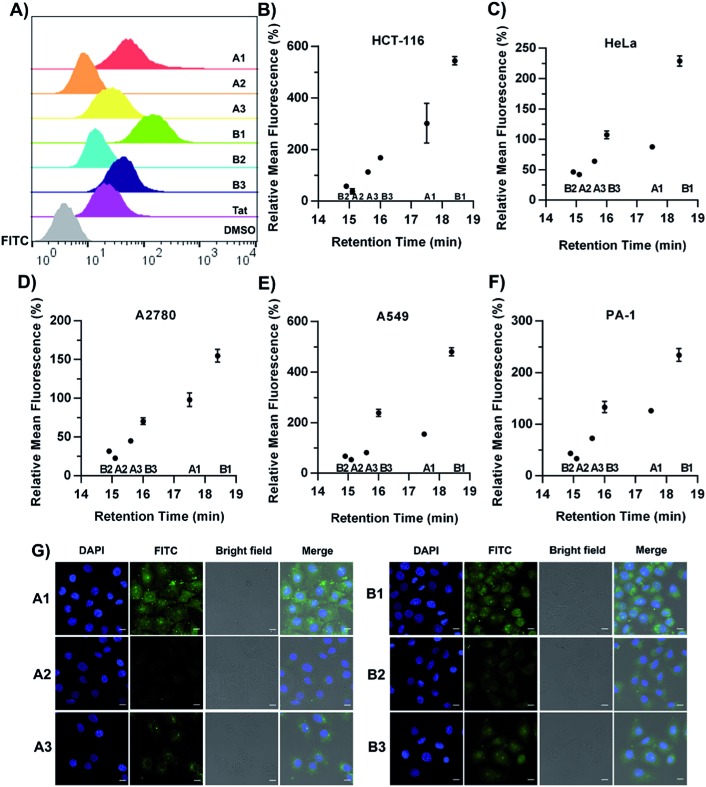
The experimental results of cell penetration in different cell lines treated with 5 μM FITC-labelled cyclic peptides using FACS analysis. (A) The representative histogram plot from flow cytometry analysis in the HCT-116 cell line. The cells were incubated with 0.05% trypan blue for 3 min prior to FACS analysis. (B–F) Plots of relative mean fluorescence *versus* retention time in the HCT-116 (B), HeLa (C), A2780 (D), A549 (E) and PA-1 (F) cell lines. The percent relative mean fluorescence, mean ± s.d., *n* = 3 and recorded data values were normalized with respect to those of the Tat peptide. (G) Confocal microscopy images of HeLa cells treated with 5 μM FITC-labelled cell-penetrating peptides (in green) for 3 hours. The nuclei were labelled with DAPI (in blue), scale bar = 10 μm.

Fluorescence-activated cell sorting (FACS) analysis was next performed to quantify the cellular uptake of the constrained peptides in different cell lines. The cells were incubated with 5 μM FITC-labelled peptides for 1 hour in a serum-free cell medium. Then, the cells were incubated with 0.25% trypsin for 10 min. In order to quench extracellular fluorescence, the cells were incubated with 0.05% (w/v) trypan blue for 3 min prior to analysis. A control experiment showed that extensive fluorescence quenching was observed when Triton-X100 was added to permeabilize the cells in the presence of trypan blue (Fig. S1†).^40^,^41^ The representative histogram plot of the cytometry assay in the HCT-116 cell line is shown in Fig. 4A. For the α-helical peptides A1–A3 with different sidechain topologies, we observed substantially different cellular uptake efficiencies in different cell lines. In the HCT-116 cell line, A1 displayed the highest cellular uptake with an 8-fold higher mean fluorescence than that of peptide A2, and peptide A3 displayed an intermediate cellular uptake between those of A1 and A2. For peptides with the β-hairpin conformation, B1 displayed a 9-fold higher mean fluorescence than B2, and peptide B3 displayed a cellular uptake between those of B1 and B2. In addition, we investigated the kinetics of the internalization of FITC-labelled A1 and A2 in HEK293T cells. The helical peptide A1 displayed faster internalization kinetics than peptide A2 (Fig. S2†). In a recent study by Walensky *et al.*, a series of stapled peptides was synthesized to determine the biophysical parameters that dictate cell penetration.^42^ They found that the calculated hydrophobicity values and HPLC retention times displayed a statistically significant relationship with the cell penetration of stapled peptides. In order to test whether our constrained peptides showed a similar relationship, we plotted the FITC intensity *versus* the retention times in different cell lines (Fig. 4B–F). Our data support the same conclusion that the internalized peptides correlate well with the HPLC retention times, suggesting that the topological distribution of the hydrophilic and hydrophobic residues dictates the overall hydrophobicity of the peptides. Additionally, there is a positive correlation between the overall hydrophobicity of the peptides and their cellular uptake efficiency, which is in accordance with previous studies.^42^ Confocal microscopy imaging was subsequently conducted to assess the cellular uptake in HeLa cells, as shown in Fig. 4G. Peptides A1/B1 showed a diffuse distribution inside the cells and peptides A2/B2 displayed significantly less fluorescence. Essentially, peptides A1 and B1 displayed substantial accumulation in the nucleus. This observation demonstrates that an optimized topology for amphipathicity is crucial for enhanced cell penetration. We propose that the membrane contacts associated with the topological distribution of the hydrophilic and hydrophobic residues could facilitate the internalization of the constrained peptides. We next performed a Cell Counting Kit-8 (CCK-8) assay to determine the toxicity of the conformationally constrained peptides. The data indicated that all of the conformationally constrained peptides do not obviously inhibit the viability of HeLa cells after 24 h of incubation at a concentration of 20 μM or 50 μM at 37 °C (Fig. S3†).

**Fig. 5:**
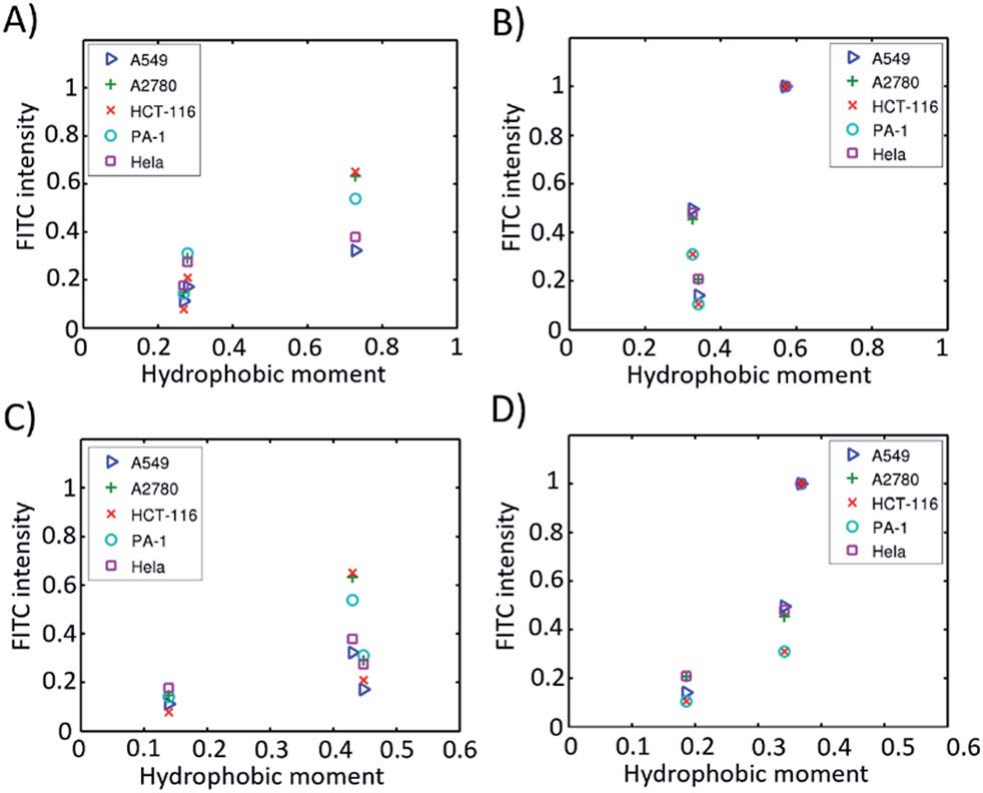
Hydrophobic moments calculated from the modelled 3D structures *versus* cell permeability in different cell lines. (A) α-Helical peptides based on eqn S2;† (B) β-hairpin peptides based on eqn (S2);† (C) α-helical peptides based on eqn (S3);† (D) β-hairpin peptides based on eqn (S3).†

Furthermore, we found that the amphipathicity of these stapled peptides characterized by the hydrophobic moment (HM) correlates well with the cellular uptake efficiency. We first performed the helix/hairpin wheel projection of these peptides and calculated the HMs based on the primary sequence from the equation 
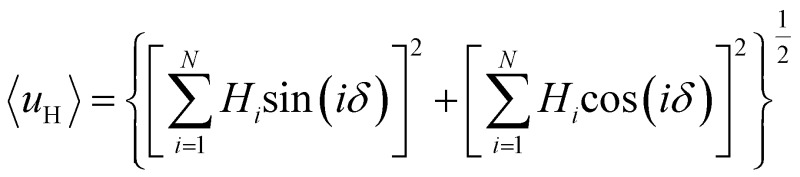
 (details in the ESI†). For the α-helix, each residue rotates 100° along the helix axis relative to the precedent residue, namely *δ* = 100°.^43^ The calculated 
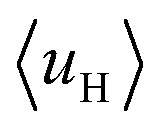
 value for the A-series peptides correlates well with the cellular uptake efficiency, as shown in Fig. S4A.† For the β-hairpin structure, *δ* is expected to range from 160° to 180°.^43^ However, we found that the calculated 
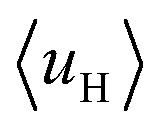
 values are sensitive to the value of *δ*. When we changed *δ* from 160° to 180°, the results differed a lot, as shown in Fig. S4B and C.† In order to accurately predict the HMs of these peptides, we further modelled the structures of these peptides using Modeller.^44^,^45^ The A-series peptides and B-series peptides were modelled as α-helix and β-hairpin conformations, respectively. Based on the modelled structures, we calculated the HMs according to the two different definitions of the HM, 
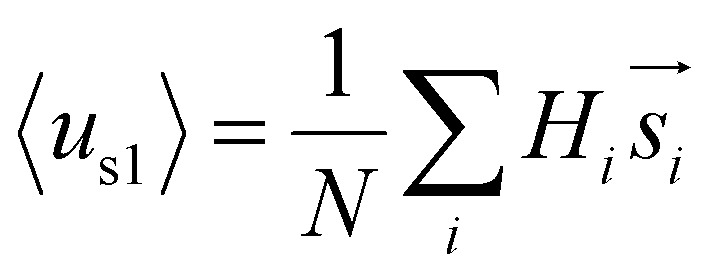
 and 
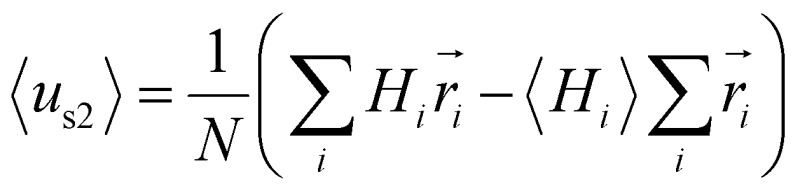
 (details in the ESI†), in which 
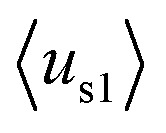
 emphasizes the amphipathicity perpendicular to the helix/hairpin axis and 
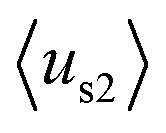
 reflects the overall amphipathicity in directions both perpendicular and parallel to the axis. Both of these results show a good correlation between the HM and the experimental cellular uptake efficiency, as shown in Fig. 5.

According to Pei’s work in 2016, the binding affinity of a peptide to the cell membrane is correlated with the cellular uptake efficiency.^23^ We hypothesized that the HM of a peptide with a well-defined secondary structure is correlated with the binding affinity. When the HM is high, the hydrophobic and hydrophilic parts of the peptide are more separated, and the hydrophobic face of the peptide will insert into the hydrophobic interior of the plasma membrane due to the hydrophobic interaction. The insertion will induce membrane curvature and the budding of the small vesicles with the peptide inside, which further accelerates the endocytosis process. When the HM is low, the hydrophobic area inserted into the plasma membrane is small, which thus decreases the hydrophobic interaction and the binding affinity resulting in a low cellular uptake efficiency. At the same time, we found that the HMs of these peptides correlate well with the retention times (Fig. S5†). This could be explained by the fact that a peptide with a larger HM will have a larger hydrophobic surface, which binds to octadecyl carbon chain-bonded silica with a larger hydrophobic interaction. The HM therefore explains the linear relation between the retention time and cellular uptake efficiency, as shown in Fig. 4B–F.

**Fig. 6:**
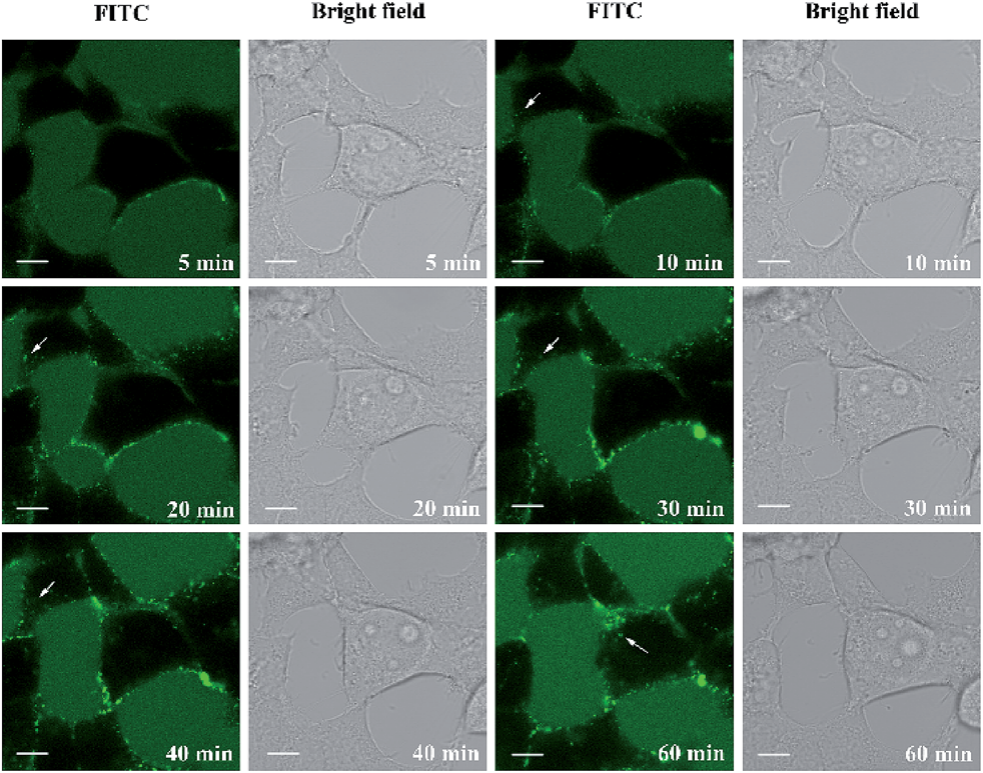
Live-cell confocal microscopic imaging of HEK293T cells treated with the 5 μM A1-avidin conjugate (in green) for indicated periods of time. The arrows (white) show the internalization of A1-avidin conjugates. Scale bar = 10 μm.

Finally, we set out to investigate the delivery capacity of these short constrained peptides as molecular transporters as a proof of principle. The most potent cyclic peptides A1 and B1 were used as model peptides. We synthesized biotinylated peptides A1 and B1. The biotin moiety was site-specifically incorporated into the N-terminus of the peptides using a PEG spacer. Commercially available FITC-labelled avidin was then pre-incubated with biotinylated peptides of equal molarity at 37 °C for 30 min to form peptide–avidin complexes through biotin–avidin interactions. Live-cell confocal microscopy imaging was then performed to monitor the internalization of the conjugates in HEK293T cells over 1 hour. The real-time monitoring of the representative peptide–avidin conjugates is shown in Fig. 6 and S6.† The incubation of HEK293 cells in the presence of 5 μM conjugates resulted in the initial accumulation of green fluorescence on the surface of the cell membrane. Spots of intense green fluorescence signals (indicated by arrows) were then observed inside the cell over time, which may indicate that the internalization of the conjugates occurred through endocytosis followed by endosomal sequestration inside the cell. Our results indicate that both A1 and B1 are capable of delivering FITC-labelled avidin into HEK293T cells. However, the punctate fluorescence in the cell periphery suggested that the protein–peptide conjugates were likely to be entrapped in the endosomes. Guanidinium-rich peptides show strong interactions with negatively charged membrane groups such as the polar heads of phospholipids or the sulfate groups of glycosaminoglycan.^46^ This process is thought to be crucial for cellular entry. For CPPs containing a low number of arginine groups or those carrying large globular cargoes, the mode of cellular uptake is restricted to endocytosis. Further sequence optimization to enhance their endosomal escape ability is now under investigation and will be reported in due course.

## Conclusions

In conclusion, we synthesized a series of constrained peptides with either α-helix or β-hairpin conformations. These short constrained peptides confer the same number of charged residues but differ in their topological distributions. Their cellular uptake efficiencies in different cell lines were tested. By modelling the structures of these constrained peptides, we calculated the amphipathicity of the peptides. We found that the amphipathicity of the peptides correlates well with the cellular uptake efficiency. We proposed that peptides with larger HMs have stronger binding affinities with the cell membrane which further accelerates the endocytosis process. We envision that this finding will shed further light on the design principle of potent conformationally constrained cell-penetrating peptides for biomedical applications.

## Conflicts of interest

There are no conflicts to declare.

## Supplementary Material

Supplementary information
