# Supplementary material for: Achieving enhanced cell penetration of short conformationally constrained peptides through amphiphilicity tuning
Source: Chem Sci. 2017 Sep 13;8(11):7576–81. doi: 10.1039/c7sc03614k (PMC5848794; doi:10.1039/c7sc03614k)
Supplement: Supplementary file 1 [file SC-008-C7SC03614K-s001.pdf]

Electronic Supplementary Material (ESI) for Chemical Science.
This journal is © The Royal Society of Chemistry 2017

Supplementary Information

Achieving Enhanced Cell Penetration of Short Conformationally Constrained Peptides through Amphiphilicity Tuning

Yuan Tian,[a, b+] Xiangze Zeng,[c+] Jingxu Li,[a+] Yanhong Jiang,[a] Hui Zhao,[a] Dongyuan Wang,[a] Xuhui Huang*[c] and Zigang Li*[a]

a. School of Chemical Biology and Biotechnology, Shenzhen Graduate School of Peking University, Shenzhen, 518055, E-mail: lizg@pkusz.edu.cn.

b. School of Life Science and Engineering, Southwest Jiaotong University, Chengdu, 610031.

c. Department of Chemistry, Center of Systems Biology and Human Health, School of Science and Institute for Advance Study, The Hong Kong University of Science and Technology, Clear Water Bay, Kowloon, Hong Kong, E-mail: xuhuihuang@ust.hk

[+] These authors contributed equally to this work.

General Information

1. Abbreviations

SPPS, solid-phase peptide synthesis; Fmoc, 9-fluorenylmethyloxycarbonyl; HCTU, 2-(1H-6-chlorobenzotriazol-1-yl)-1,1,3,3-tetramethyluronium hexafluorophosphate; DIPEA, diisopropylethylamine; DMF, dimethylformamide; ESI-MS, electrospray ionization mass spectrometry; MBHA, 4-methyl-benzylhydrylamine; RP-HPLC, reserved-phase high performance liquid chromatography; RT, room temperature; TFA, trifluoroacetic acid; TFE, 2,2,2-trifluoroethanol; TIS, triisopropylsilane; Et_2O, diethyl ether; LC-MS, liquid chromatography–mass spectrometry; HPLC, high-performance liquid chromatography;

2. Materials

All solvents and reagents used for solid phase peptide synthesis were purchased from Shanghai Hanhong Chemical Co., J&K Co. Ltd., Shenzhen Tenglong Logistics Co. or Energy Chemical Co. and were used without further purification unless otherwise stated.

3. HPLC and Mass spectrometry

Peptides were analyzed and purified by HPLC (SHIMAZU Prominence LC-20AT) with a C18 analytic column (Agilent ZORBAX SB-Aq, 4.6 × 250 mm, 5 μm, flow rate 1.0 mL/min). ddH_2O (containing 0.1% TFA) and pure acetonitrile (containing 0.1% TFA) were used as mobile phase. LC-MS data were carried out on SHIMAZU LC-MS 8030 (ESI-MS).

4. Peptide Synthesis and Characterization

Peptide synthesis was performed manually on Rink Amide MBHA resin (loading capacity: 0.54 mmol/g) by standard Fmoc-based SPPS. Generally, Rink amide AM resin was pre-swelled with DMF for 1 hour. Fmoc deprotection was performed with 50% morpholine (in DMF) for 30min × 2. Then the resin was washed with DMF (5 times), DCM (5 times) and DMF (5 times). Fmoc-protected amino acids (5.0 equiv according to initial loading of the resin) and HCTU (4.9 equiv) were dissolved in NMP, followed by DIPEA (10.0 equiv). The mixture was pre-activated for 1 min and added to the resin for 2 hours, then the resin was washed with DMF (5 times), DCM (5

times) and DMF (5 times). For FITC labeling, the resin was treated with a mixture of FITC (5.0 equiv according to initial loading of the resin) and DIPEA (10.0 equiv) dissolved in DMF in dark overnight. After that, peptides were cleaved from the resin with a mixture of TFA/H_2O/EDT/TIS (94:2.5:2.5:1) for 2 hours and concentrated under a stream of nitrogen. The crude peptides were then precipitated with Hexane/Et_2O (1:1 in volume) at -20 ℃, isolated by centrifugation then dissolved in water/acetonitrile, purified by HPLC and analyzed by LC-MS. Analytical data are shown in Supporting Table S1. FITC labeled peptides were quantified by absorbance of 494nm with an extinction coefficient of 77,000 M^{-1} cm^{-1}.

Experimental section

1. Representative synthetic scheme of conformationally constrained A1 and B1.

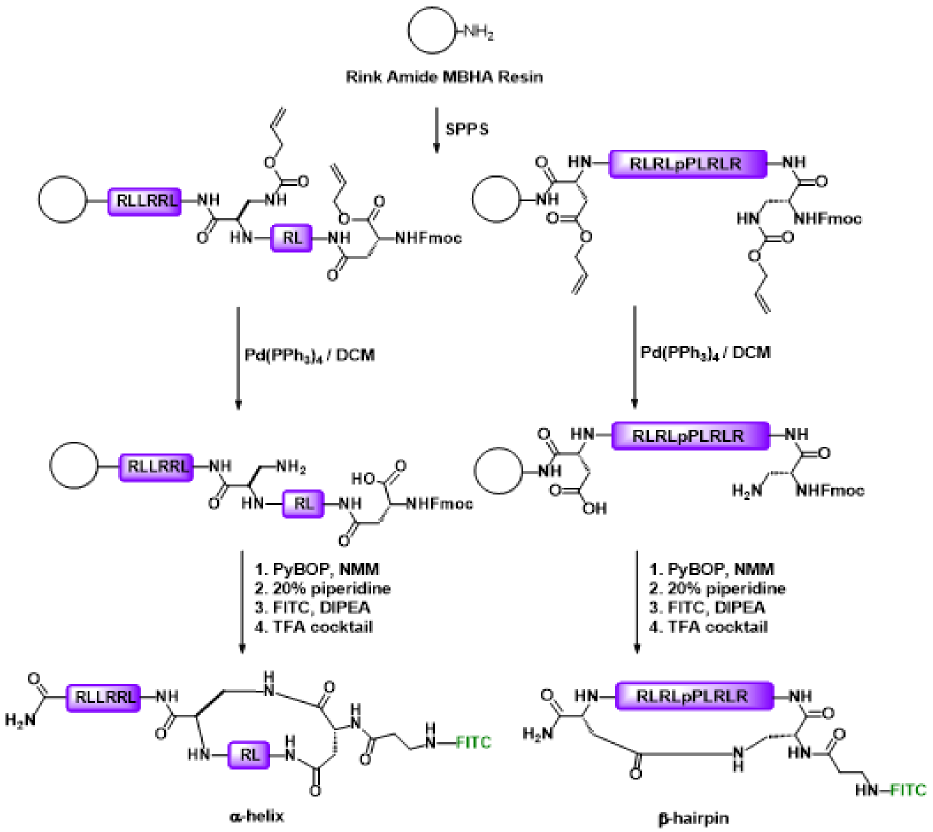

SPPS was performed by standard protocol mentioned in general information. The allyl ester and allyl carbamate protection groups were removed using $Pd(PPh_3)_4$ (0.1 equiv) and N,N-dimethylbarbituric acid (4.0 equiv), in DCM for 2 hours × 2. Macrolactamization was performed on the resin using PyBOP/HOBt/NMM (2.0 : 2.0 : 2.5) in DMF. After FITC labeling and cleavage from the resin, crude peptides were purified by RP-HPLC mentioned above with a linear gradient of 5 to 60% acetonitrile in 40 min.

2. Circular Dichroism Spectroscopy (CD)

CD spectra were obtained using a Chirascan Plus Circular Dichroism Spectrometer at 20 ˚C. Peptides were dissolved in 10 mM sodium phosphate buffer (pH 7.4) at concentrations of 50 μM. Parameters used in the experiment are as followed: wavelengths from 250 to 190 nm were measured with resolution of 0.5 nm, response of 1 s, bandwidth of 1 nm, scanning speed of 20 nm/min. CD data are presented as mean residual elipticity [θ] in deg cm^2 $dmol^{-1}$. Concentrations were determined as mentioned in general information.

3. Flow Cytometry Analysis

A549, A2780, HCT-116, PA-1, HeLa cells were seeded in 24 well culture plate. The plates were incubated with FITC-labeled peptides at concentration of 5μM at 37 °C in fetal bovine serum (FBS)-free cell medium (For A549 and A2780 cells, RPMI Medium 1640 basic (Lot. 8116264) was used; For HeLa cells, DMEM cell medium (Lot. 1868775) was used; For HCT-116 cells, McCoy's 5A Medium (Lot. 1835937) was used; For PA-1 cells, MEM (Lot. 1827455) was used; Cell medium was purchased from Gibco.) in the presence of 5% CO_2. After 1hour incubation, peptide containing medium was removed followed by washing with phosphate buffered saline (PBS). The cells were then digested with 0.25% trypsin for 10 min. After that, cells were incubated with 0.05% Trypan Blue for 3 min. Then the cells were analyzed by flow cytometry (FACSCalibur™). A minimum of 10,000 gated events were acquired and analyzed. Experiments were performed in triplicate. For control experiment for Trypan Blue assay, cells were permeabilized with 0.05% (v/v) Triton-X100 and fluorescence was analyzed by flow cytometry.

4. Confocal Microscopy Imaging

Hela Cells were seeded on the coverslips and then incubated with 5μM FITC-labeled peptides for 3 hours. After that, peptide containing media was removed followed by washing with phosphate buffered saline (PBS). Then the cells were fixed with 4% paraformaldehyde for 15 min. Nuclei were stained with 4',6-diamidino-2-phenylindole (DAPI) for 5 min then washed with PBS. The coverslips were mounted onto slides and visualized by confocal laser scanning microscope.

5. Live Cell Confocal Microscopy Monitoring of Protein Conjugate Internalization

HEK293T Cells were seeded on the Glass Bottom Cell Culture Dish (Nest, Φ 15mm). Commercially available FITC labelled avidin was purchased from the invitrogen (Lot: 43-4311). For the preparation of peptide-avidin complex, FITC labelled avidin was pre-incubated with equal molar biotinylated peptides at 37°C for 30 min to form peptide-avidin complex through biotin-avidin interactions. After that, the mixture was added to the dish and visualized by confocal laser scanning microscope for real time monitoring.

6. Cell viability

Hela cells were seeded in 96-well plates with 5000 cells per well and incubated under 37℃ for 12 h. After that, cell media was removed followed by adding 20μM of peptides to the 96-well plates and incubate for 24h. Then the media was replaced by CCK8 reagent (Solarbio, Lot: CA1210) and incubated for another 1h. The absorbance was scanned by a microplate reader (Perkin Elmer, Envision, 2104 Multilabel Reader). Wavelength, 450nm.

7. Calculation of Hydrophobic moment (HM)

In this work, we calculated HM of these stapled peptides based on both their primary sequences and modeled 3D structures [1-3].

7.1. Based on the primary sequence

In this method, the helix peptide will be projected onto the plane perpendicular to the helix axis, namely represented by the helical wheels. Then, the following equation is applied to calculate HM:

$$\langle u_H \rangle = \{[\sum_{i=1}^{N} H_i \sin(i\delta)]^2 + [\sum_{i=1}^{N} H_i \cos(i\delta)]^2\}^{\frac{1}{2}} \quad \text{(S1)}$$

In which N is the number of residues in the peptide, H_i is the hydrophobicity of ith residue defined in Eisenberg's work [3]. Two adjacent residues differ by 100 degree in the plane perpendicular to the helix axis, namely, $\delta = 100°$. Similarly, we could project the beta-hairpin peptides onto the plane perpendicular to the hairpin axis and calculate HM. For typical beta strand structures, two residues differ by $160°$ ~ $180°$.

7.2. Based on 3D structures

a. Definition I

$$\langle u_{s1} \rangle = \frac{1}{N} \sum_i H_i \vec{s_i} \quad \text{(S2)}$$

In which N is the number of residues in the peptide, H_i is the hydrophobicity of ith residue, $\vec{s_i}$ is the unit vector from the C_α atom to the mass centre of the side chain of the ith residue.

b. Definition II

$$\langle u_{s2} \rangle = \frac{1}{N} \left(\sum_i H_i \vec{r_i} - \langle H_i \rangle \sum_i \vec{r_i} \right) \quad \text{(S3)}$$

In which $\vec{r_i}$ is a vector from the mass center of the peptide to the mass center of the side chain of ith residue.

Supporting Figures

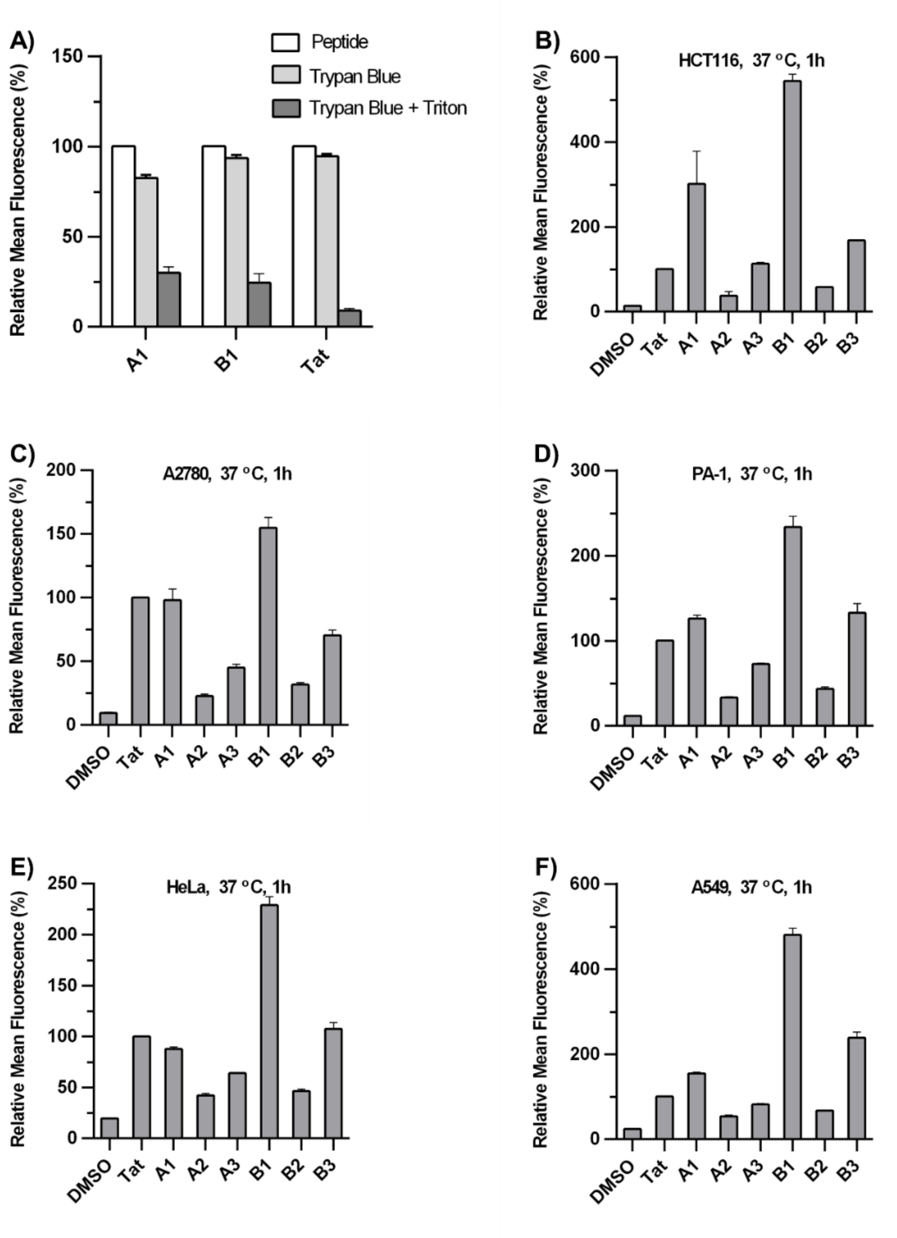

Fig. S1. Flow cytometry analysis of different cell-lines treated with conformationally constrained peptides. A) Flow cytometry analysis of HCT116 cells treated with A1, B1, and Tat peptides in the presence and absence of Trypan Blue and Triton-X100. B-F) Flow cytometry analysis of HCT116 cells (B), A2780 cells (C), PA-1 cells (D), HeLa cells (E), A549 cells (F) treated with 5 μM fluorescently labelled compounds at 37 °C for 1 h. Cells were incubated with 0.05% Trypan Blue for 3 min prior to FACS analysis. Percent relative mean fluorescence, mean ± s. d., n=3 and values are normalized with respect to Tat peptide.

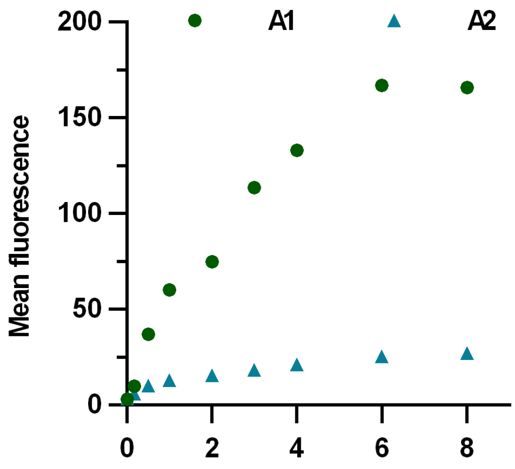

Fig. S2. Kinetic study of the internalization of FITC labelled peptide A1 and A2 at concentration of 5µM in HEK293T cells at 37 ^{o}C.

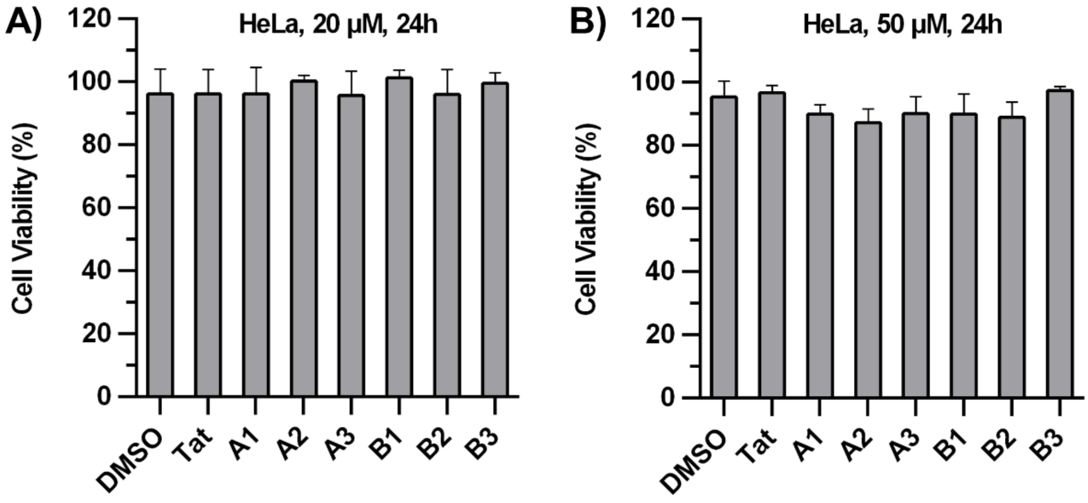

Fig. S3. Cytotoxicity of different conformationally constrained peptides as monitored in HeLa cells. Cell viability was determined using the CCK-8 assay after 24 h of incubation at a concentration of 20 μM (A) or 50 μM (B) at 37 °C. DMSO treated control cells were normalized to 100% cell viability. Percent cell viability, mean ± s. d., n=4.

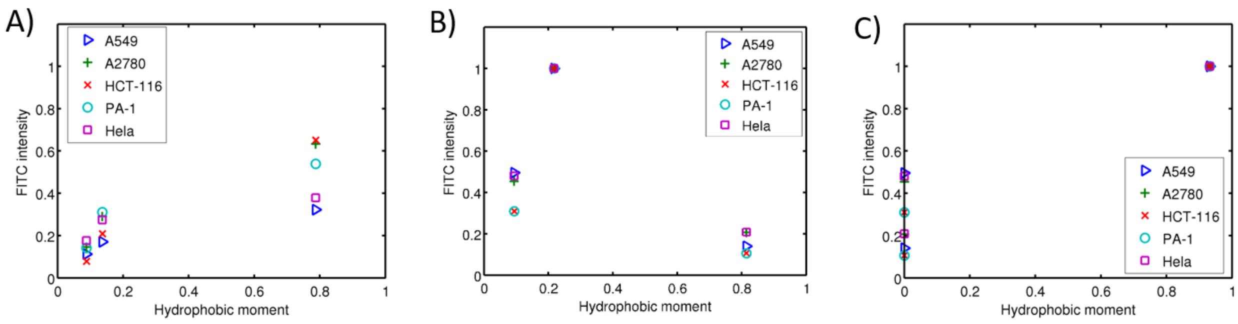

Fig. S4. Hydrophobic moment calculated from the primary sequence versus cell permeability in different cell lines: A) alpha helix peptides; B) beta hairpin in which two adjacent residues differing by 160 degree; C) beta hairpin in which two adjacent residues differ by 180 degree.

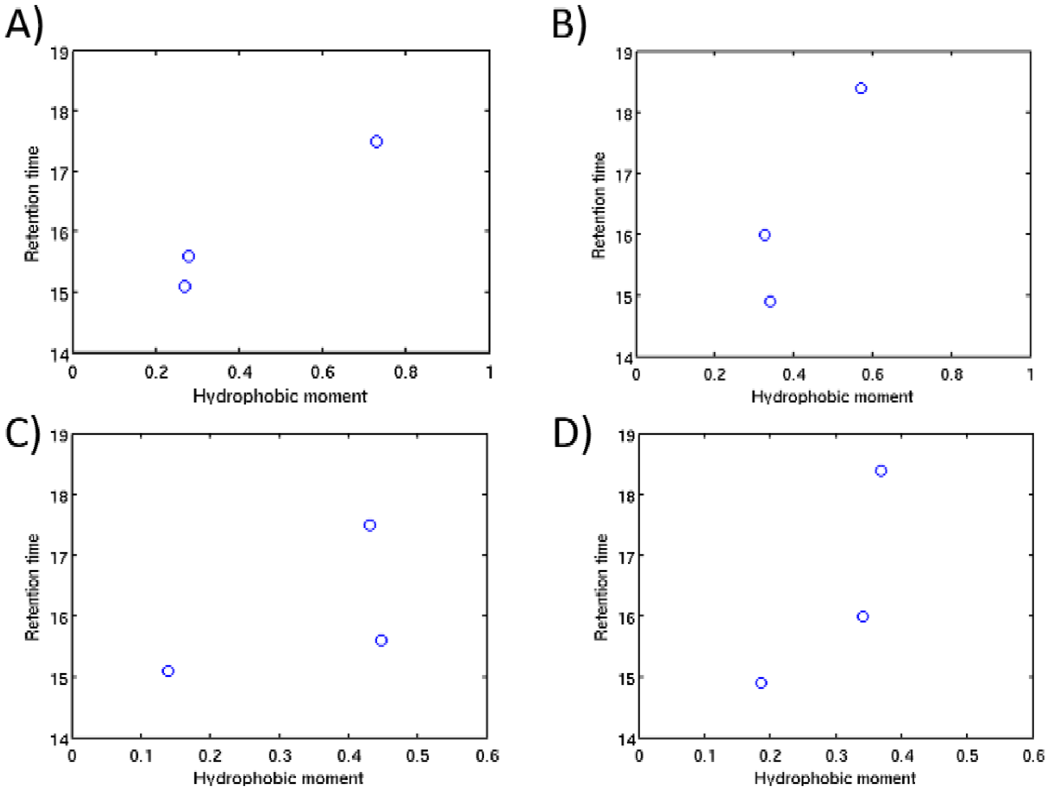

Fig. S5. Hydrophobic moment calculated from the modeled structures versus retention time. A) A peptides based on Eq. S2 ; B) B peptides based on Eq. S2. C) A peptides based on Eq. S3 ; D) B peptides based on Eq. S3.

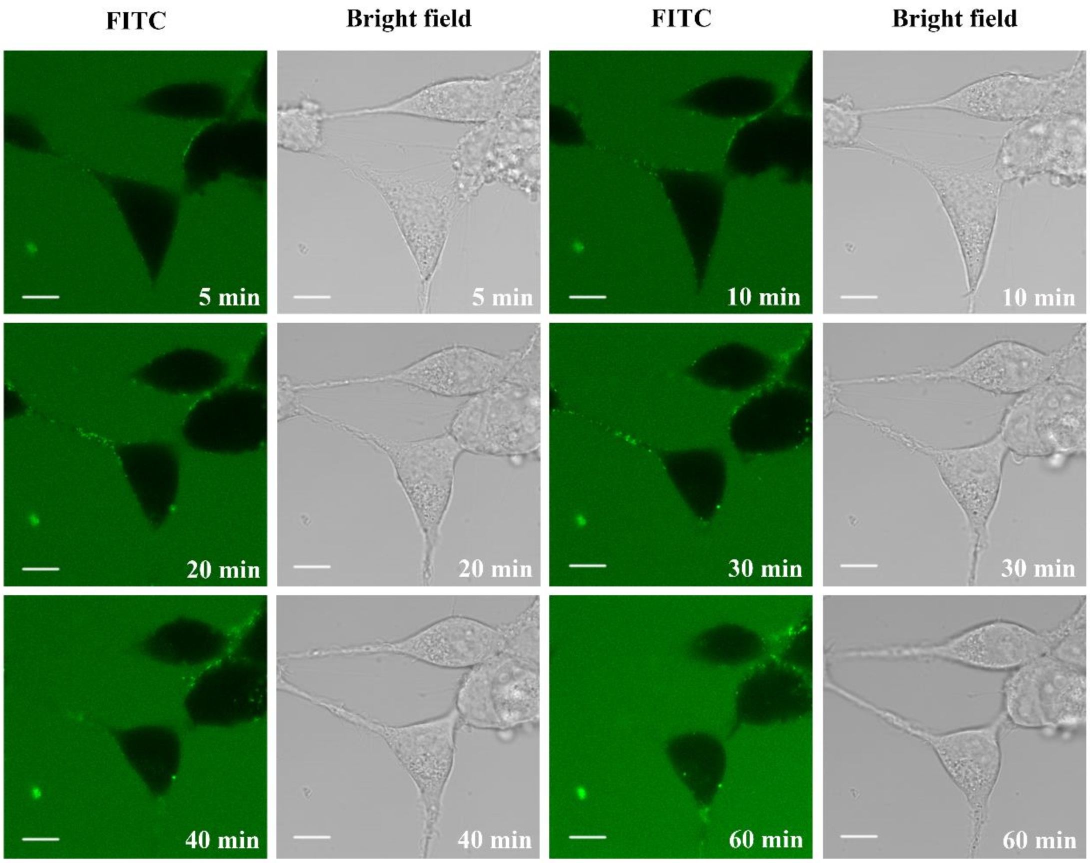

Fig. S6. Live-cell confocal microscopic imaging of HEK293T cells treated with 5μM B1-avidin conjugate (in green) for indicated periods of time. Scale bar, 10 μm.

Appendix

HPLC Traces and MS Spectra

A1 FITC-βA-(cyclo-1, 4)-[isoAspLRDapLRRLLR]-NH_2

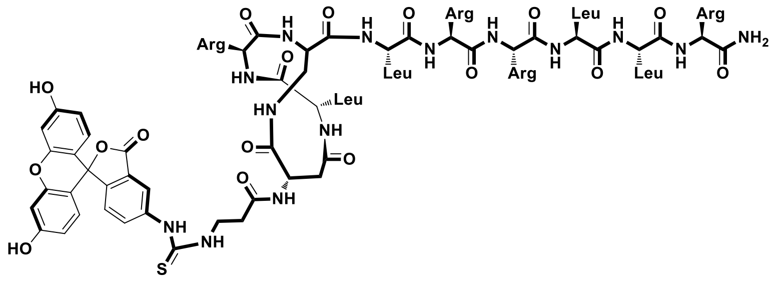

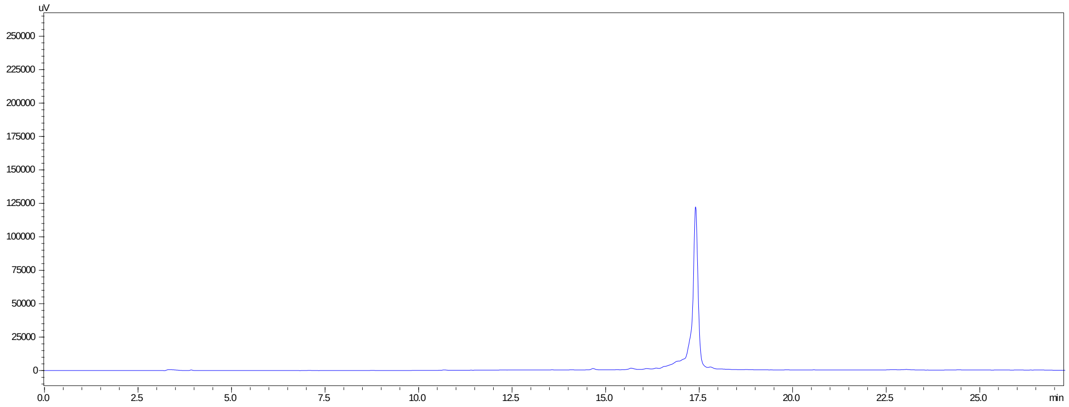

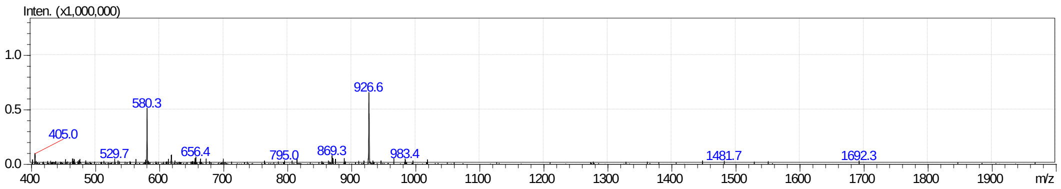

A2 FITC-βA-(cyclo-1, 4)-[isoAspLRDapRLLRLR]-NH_2

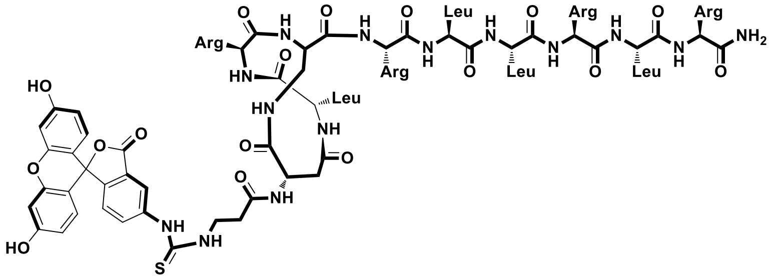

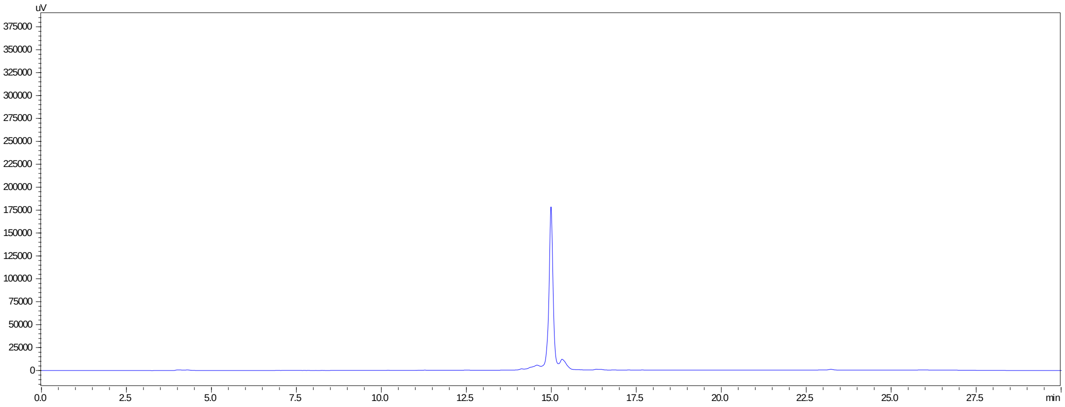

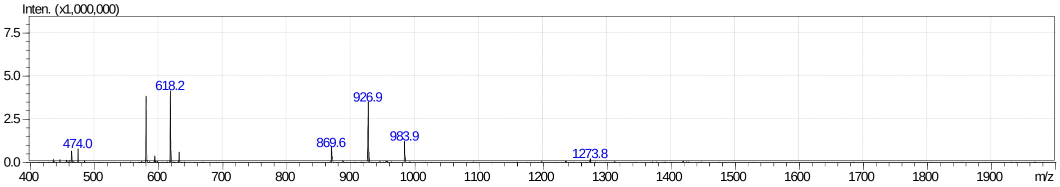

A3 **FITC-βA-(cyclo-1, 4)-[isoAspRLDapRRLLLR]-NH₂**

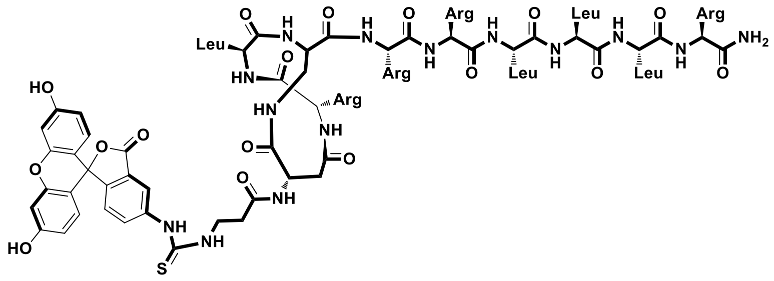

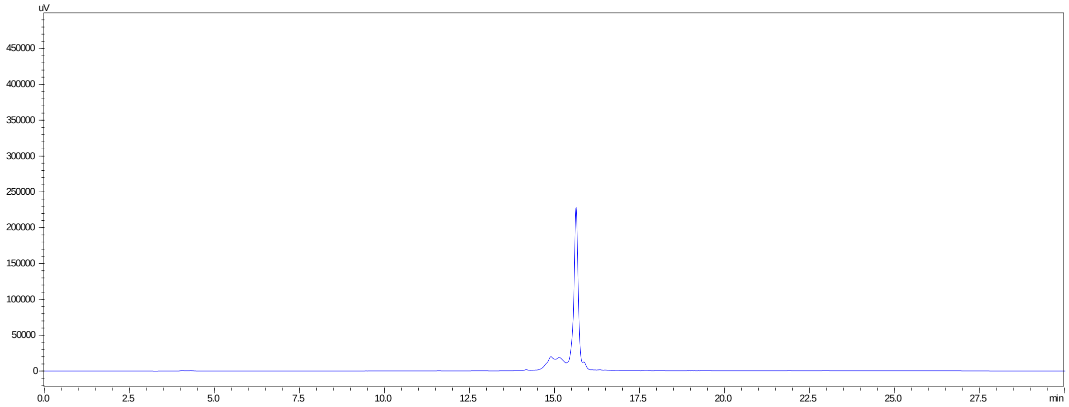

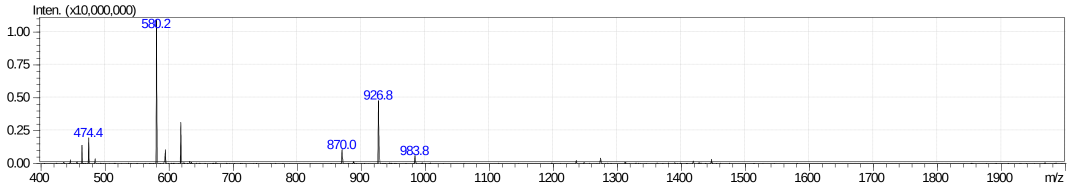

B1 FITC-βA-(cyclo-1, 12)-[DapRLRLpPLLLLAsp]-NH_2

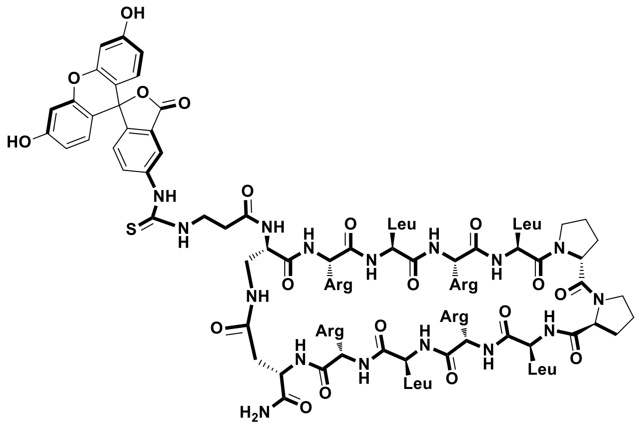

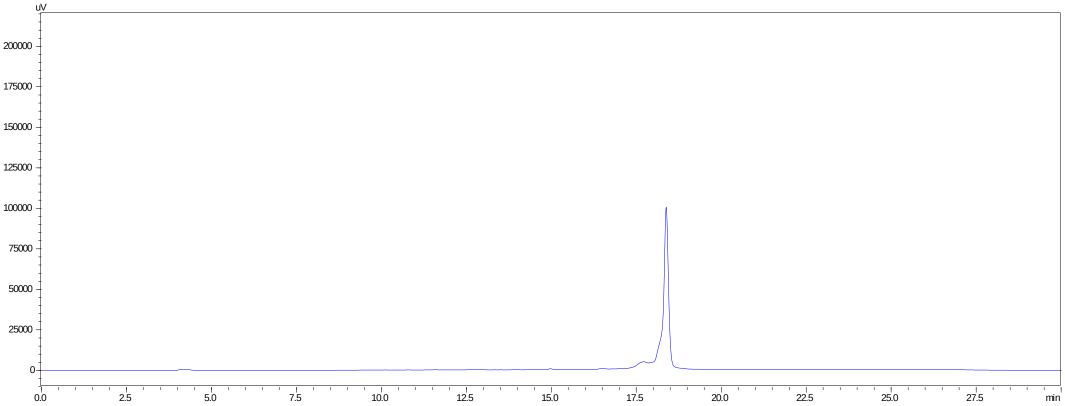

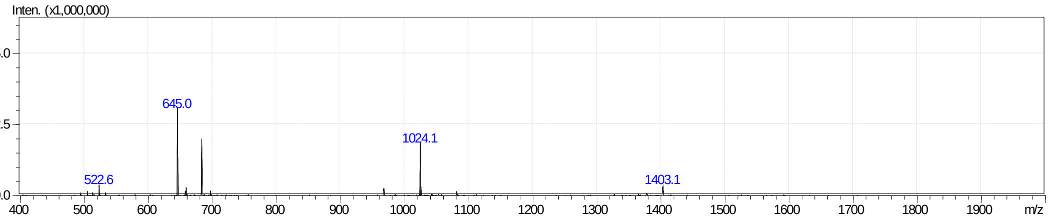

B2 FITC-βA-(cyclo-1, 12)-[DapLRLRpPLRLRAsp]-NH_2

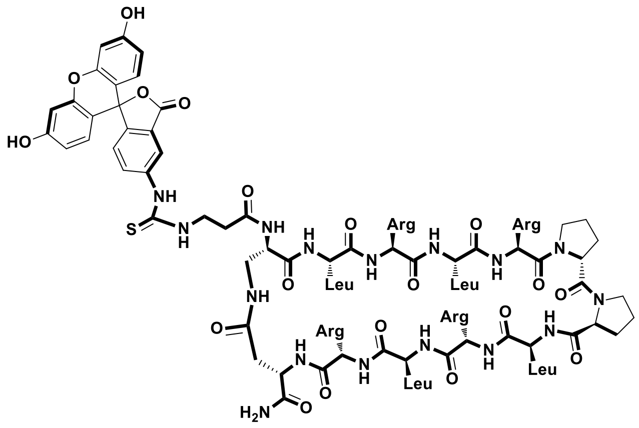

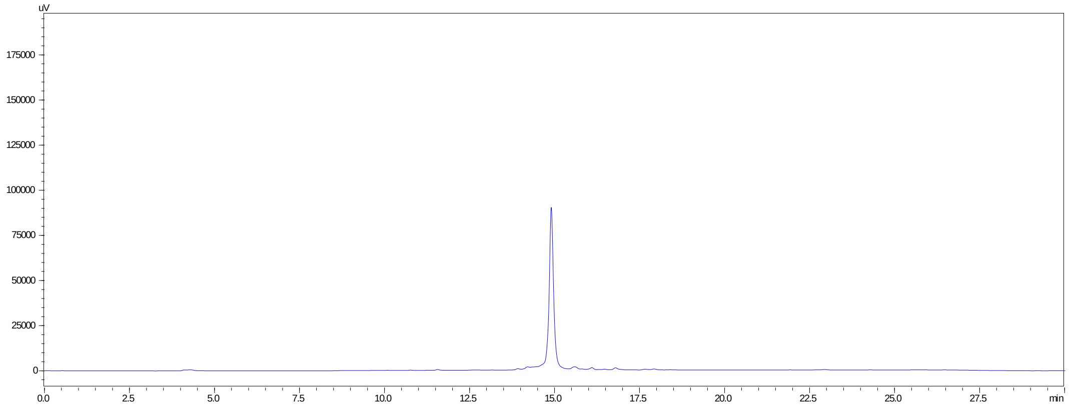

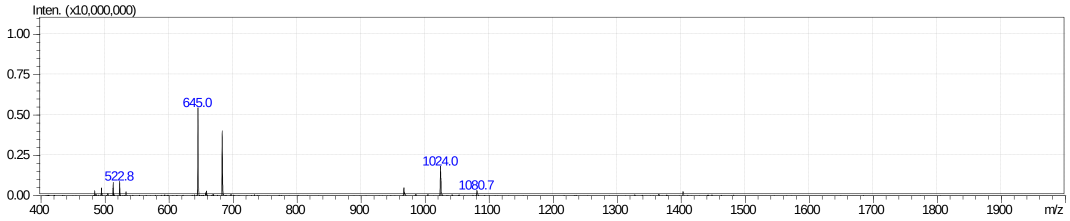

B3 **FITC-βA-(cyclo-1, 12)-[DapRRRRpPLLLLAsp]-NH₂**

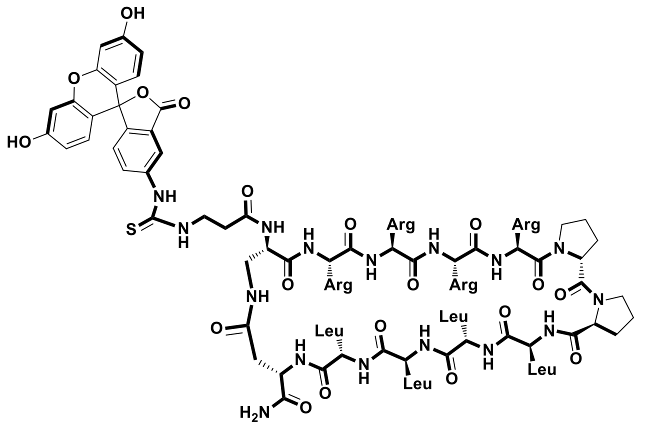

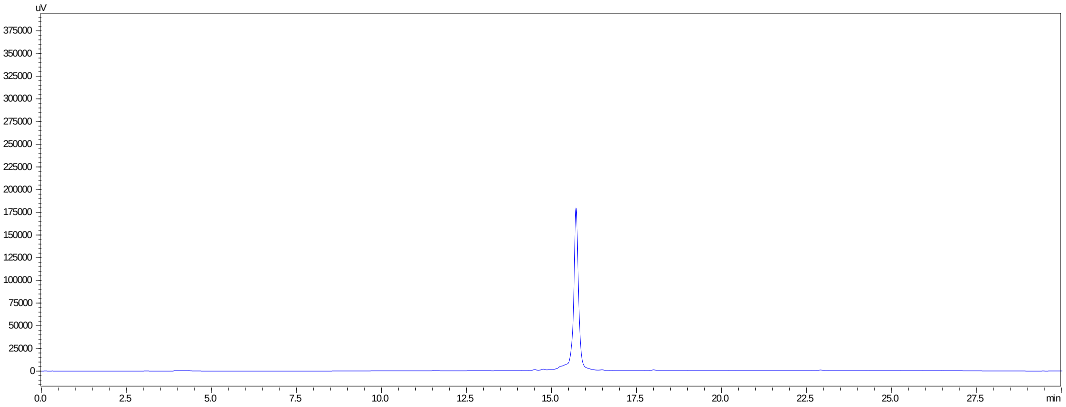

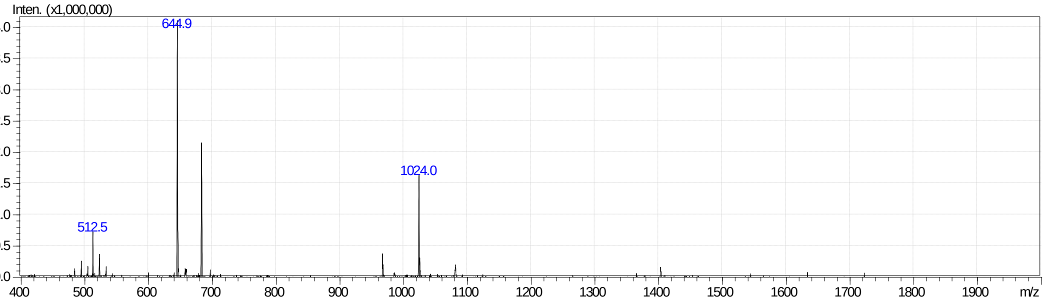

Tat

FITC-βA-RKKRRQRRR-NH_2

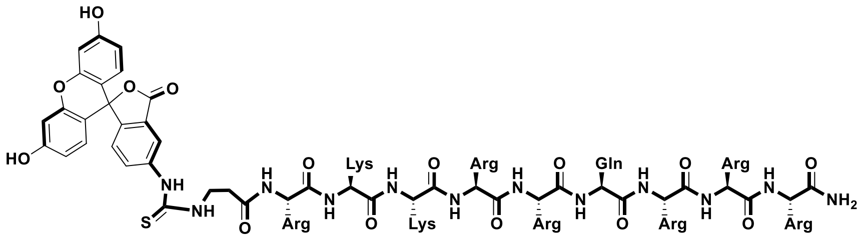

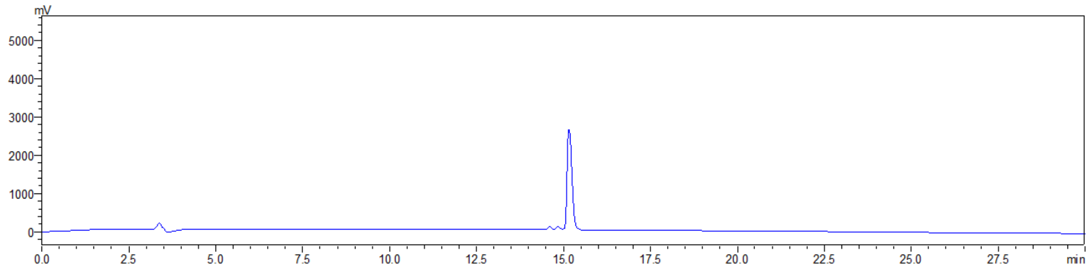

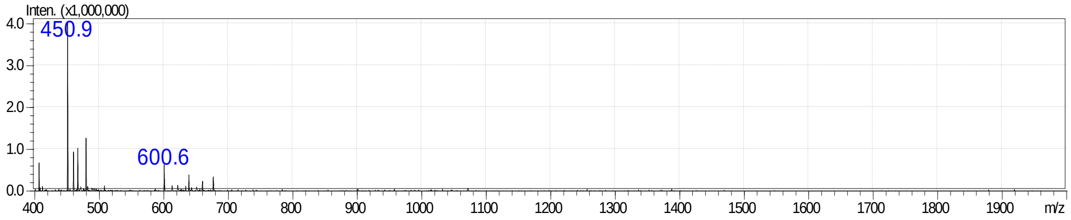

Reference

1. Eisenberg, D., R.M. Weiss, and T.C. Terwilliger, *The helical hydrophobic moment: a measure of the amphiphilicity of a helix*. *Nature*, 1982. **299**(5881): p. 371-374.
2. Eisenberg, D., R.M. Weiss, and T.C. Terwilliger, *The hydrophobic moment detects periodicity in protein hydrophobicity*. *Proceedings of the National Academy of Sciences*, 1984. **81**(1): p. 140-144.
3. Eisenberg, D., et al. *Hydrophobic moments and protein structure*. in *Faraday Symposia of the Chemical Society*. 1982. Royal Society of Chemistry.
